# Association between Fresh Fruit and Vegetable Consumption and Purchasing Behaviors, Food Insecurity Status and Geographic Food Access among a Lower-Income, Racially/Ethnically Diverse Cohort in Central Texas

**DOI:** 10.3390/nu14235149

**Published:** 2022-12-03

**Authors:** Kathryn M. Janda, Nalini Ranjit, Deborah Salvo, Aida Nielsen, Catherine Kaliszewski, Deanna M. Hoelscher, Alexandra E. van den Berg

**Affiliations:** 1 Department of Public Health, Robbins College of Health and Human Sciences, Baylor University, Waco, TX 76798, USA; 2 Department of Health Promotion and Behavioral Science, School of Public Health in Austin, University of Texas Health Science Center in Houston in Austin, Austin, TX 78701, USA; 3 Michael and Susan Dell Center for Healthy Living, Austin, TX 78701, USA; 4 Department of Kinesiology and Health Education, University of Texas at Austin, Austin, TX 78712, USA

**Keywords:** food insecurity, geographic access to food, fresh fruit and vegetable consumption, fresh fruit and vegetable purchasing behaviors, disparities, intersectionality

## Abstract

The aims of this study were to determine if fresh fruit and vegetable consumption and purchasing behaviors were associated with geographic food access and/or food insecurity status, and to explore the role of sociodemographic characteristics among participants of a lower-income, racially/ethnically diverse cohort. This study used a cross-sectional design and baseline survey data from the FRESH-Austin study (N = 393). Associations between fresh produce consumption/purchasing and food insecurity status and geographic access to food were assessed utilizing univariate, bivariate, and multivariate linear regression methods and potential interactions were examined. The sample 40% reported being food insecure and the majority identified as Hispanic. Geographic food access was directly associated with fresh produce consumption (β = 0.46, *p* = 0.02); however, the directionality of the relationship between food insecurity and fresh produce consumption varied due to a significant interaction with race/ethnicity. Only utilizing food assistance was associated with purchasing fewer fresh produce (β= −1.83, *p* = 0.03). Findings suggest that communities experience food insecurity and limited healthy food access in different ways, and in some situations, are associated with fresh produce consumption and purchasing behaviors. Future research adopting an intersectionality-sensitive approach to better understand how to best support communities at risk is needed.

## 1. Introduction

### 1.1. Food Insecurity and Geographic Access to Food Issues and Disparities

Food insecurity occurs when individuals lack stable availability or the ability to acquire safe and nutritional foods in a socially appropriate or acceptable manner [1]. Food insecurity is prevalent in the United States with the prevalence of food insecurity at 11.1% among American households in 2018 and 10.5% of American households in 2019 according to United States Department of Agriculture, Economic Research Service data [2]. The prevalence of food insecurity varies by state, with Texas having a prevalence that exceeds the national average with approximately 14% of Texas families experiencing food insecurity in 2018 [2]. Additionally, the prevalence of food insecurity is higher than the national prevalence in specific metropolitan areas in the state of Texas, such as the greater Austin/Travis County area with 12.8–12.9% of households identifying as food insecure in 2019 [3]. This high prevalence of food insecurity is a public health concern, given that poor diet and health conditions such as undernutrition, anemia, obesity, diabetes, hypertension, and others have all been found to be directly associated with food insecurity [4,5,6,7]. Therefore, it is incredibly important to the advancement of field of public health nutrition and community health to further investigate food insecurity, various indicators, and how food insecurity could impact behaviors associated with chronic disease development, such as dietary behaviors [8,9,10,11].

Food insecurity is conceptualized through four pillars: availability, access, utilization, and stability over time [1,12]. Access is framed as having food geographically proximal, economically attainable, and culturally relevant foods that are able to be obtained with relative ease. When examining food insecurity, geographic access to food is one of the most commonly discussed, researched, and intervened upon components discussed in the literature [13,14,15,16,17,18,19]. However, while they are conceptually tied, the association between the two is varied. Some researchers have found that geographic access and food insecurity were associated, with individuals experiencing limited geographic access to food having a greater likelihood of experiencing food insecurity; however, these findings are not always consistent across samples and settings in the US [14,18,20,21]. In an effort to elucidate these relationships, it is imperative to address the intersectionality of these exposures to create a comprehensive understanding of associated factors that are driving behaviors within communities.

### 1.2. Association between Food Insecurity, Geographic Access to Food and Fruit and Vegetable Consumption and Purchasing Behaviors: Gaps in the Literature

Consuming a low-quality diet, specifically lower fruit and vegetable consumption than the recommended dietary guidelines, is one notable behavior that has been found to be associated with food insecurity and limited geographic access to food in the literature. For instance, food insecure households, as well as individuals living in lower-income communities and lower-income households, have a greater likelihood of having a diet with reduced fruit and vegetable intake [22,23,24,25]. Additionally, there have been mixed findings on the association between race/ethnicity and fruit and vegetable consumption, with some studies stating that racial-ethnic minorities have a lower likelihood of meeting dietary recommendations for fruit and/or vegetable consumption [10,26,27]. However, work by numerous scholars examining nationally representative data and context-specific cohorts found Hispanic participants had a greater likelihood of meeting fruit and vegetable consumption recommendations compared to non-Hispanic white participants, while Black participants had a lower likelihood of meeting fruit and vegetable consumption recommendations compared to non-Hispanic white participants [23,28,29,30]. These disparities are particularly concerning given the impact of food insecurity and low-quality diet on health, as mentioned previously. However, a commentary by Houghtaling and colleagues (2022) has called for greater exploration of the role of intersectionality, meaning the converging influence of multiple sociodemographic factors that simultaneously exist within an individual [31], and fruit and vegetable consumption [8].

Many researchers and policy advocates have examined and emphasized the role of physical geographic access to food, particularly to large retailers such as supermarkets and large grocery stores, as a key indicator of healthy food access and healthy eating behaviors [17,32,33,34,35]. However, the literature demonstrating the impact of introducing new grocery stores into communities on dietary behaviors has had mixed findings [14,34,36,37,38]. Specifically, with some studies finding that healthy eating behaviors improved with the introduction of new grocery stores; however, other studies have found that healthy and unhealthy dietary behaviors increased with the introduction of a new grocery store findings [14,34,36,37,38]. Furthermore, there has been limited exploration of the impact of food insecurity and geographic food access on fruit and vegetable purchasing behaviors. The limited work that has been done has mainly explored shopping behaviors such as store selection, shopping motivations, and frequency of grocery shopping trips rather than exploring what items were purchased [21,39]. A scoping review by Singleton and colleagues (2020) examined studies that evaluated consumer food purchasing in the U.S. with an emphasis on an intersectional approach [11]. In their review, they found that 34 studies examined food purchasing behaviors; however, only three studies specifically examined fruit and vegetable purchasing and had an intersectional approach, and only incorporated race/ethnicity and socioeconomic status into the analysis [11,40,41]. Thus, further work is needed to examine how food insecurity, geographic access to food, and various sociodemographic factors are associated with fresh fruit and vegetable consumption and purchasing behaviors utilizing an intersectional approach.

### 1.3. Purpose of the Study

The objective of this study was three-fold: (1) to determine if fresh fruit and vegetable consumption of participants of a lower-income, racially/ethnically diverse cohort were associated with geographic food access and/or food insecurity status, (2) to determine if fresh fruit and vegetable purchasing behaviors of participants of a lower-income, racially/ethnically diverse cohort were associated with geographic food access and/or food insecurity status and (3) explore the potential moderating role of sociodemographic characteristics (race/ethnicity, income, urbanicity, and food assistance-related factors) as potential moderators in the association between fresh fruit and vegetable consumption/purchasing and geographic food access and/or food insecurity status.

## 2. Materials and Methods

### 2.1. Overview of Sample and Parent Study

This study utilized a cross-sectional study design, conducting secondary data analysis with baseline survey data from the FRESH-Austin study, which has been previously described in great detail in Janda et al., 2021 [42]. One component of the FRESH-Austin study was a cohort (N = 400) study in which participants were recruited from Eastern Travis County. Participants were purposefully recruited in three ways: (1) 130 participants were recruited via random intercept surveys at participating FRESH retail assets; (2) 185 participants were recruited via door-to-door from a sample randomly selected street segments within a 1.5-mile street network buffer of participating FRESH assets, and; (3) 85 participants were recruited door-to-door from a sample of randomly selected street segments in comparison neighborhoods which were deemed comparable and similar to the communities near participating FRESH assets based on sociodemographic characteristics from 2017 American Community Survey data [43].

Cohort participants completed a baseline survey between October 2018-March 2019, were adults aged 18 or older, identified as the primary food shopper for the household, were fluent and able to speak English or Spanish, and provided a home address at the time of data collection. The analytic sample used for this study restricted the original sample to only those residing in Travis County and thus included 393 cohort participants. Additionally, the City of Austin’s Food Environment Analysis (FEA) provided data regarding food retail location data.

### 2.2. Independent Variables of Interest: Food Insecurity and Geographic Access to Food Measures

For this analysis, geographic food access and food insecurity were the independent variables and exposures of interest. Geographic food access was measured in two ways: (1) through a binary variable highlighting the presence of a supermarket within a 1500 m network buffer, and (2) a continuous variable that measured how far the participant traveled to their self-reported grocery store measured in miles. Network buffers utilized street networks that had a 1500 m radius centered around each cohort participant’s home address and were developed in ArcGIS, with the distance being informed by commonly used cut-points for “walkable distances” in the literature [44,45]. Using data from the City of Austin, Office of Sustainability’s Food Environment Analysis of Travis County, a food retail environment layer was created to analyze food retail location data by geocoding all grocery stores/supermarkets from their dataset [46]. Subsequently, binary variables were created to determine the presence of supermarkets/large grocery stores that the participant reported shopping at within the 1500 m network buffers.

The measure assessing how far a participant traveled to their preferred grocery store resulted in a continuous variable measuring the street network distance (in miles) between the participant’s home and self-reported utilized supermarket/large grocery store. This variable was created using a combination of software and approaches, such as ArcMap, R, and Google Distance Matrix API [44,47]. The specific stores were self-reported by the respondent in the FRESH-Austin baseline survey and the addresses of reported stores geocoded. Supermarkets/large grocery stores were selected as the type of food retail of interest for this analysis given that the entirety of the sample reported shopping at supermarkets (100%).

As previously stated, the second independent variable for this analysis was food insecurity status. Food insecurity status was measured using the 2-item food insecurity screener that was present in the baseline FRESH-Austin survey. This validated food insecurity measure asks respondents if the following two statements: “1. We worried our food would run out before we got money to buy more.”; “2. The food we bought just didn’t last and we didn’t have money to get more.” were experiences that were “Often True,” “Sometimes True,” or “Never True” in the last year [48,49,50]. Participant answers to these two questions were then then dichotomized as food secure and sometimes/often food insecure, as is common in the literature to food secure vs. sometimes/often food insecure [48,49,50].

### 2.3. Dependent Variables of Interest: Fresh Fruit and Vegetable Consumption and Purchasing Measures

Fresh fruit and vegetable consumption was measured using the FRESH Food Frequency Questionnaire (FFQ), a modified Block food frequency questionnaire that had been validated using 24-h dietary recalls prior to baseline data collection [42,51]. The validation of the FRESH FFQ has been described at length by Jovanovic et al. 2021, but was adapted from a Block food frequency questionnaire in another study conducted in Central Texas among a predominantly Hispanic sample of Supplemental Nutrition Assistance Program (SNAP) participants [51,52]. Given the focus of the parent study was on fresh produce, there were numerous fruits and vegetables included in the FRESH FFQ, including: carrots, lettuce, avocados, dark leafy greens, broccoli/cauliflower, peppers, tomatoes, sweet potatoes, potatoes (not sweet), corn, cabbage, zucchini/other squash, onions, apples, berries, citrus, grapes, bananas, melon, and up to four additional vegetables and fruits the consumed outside of this list. For each of these types of fresh produce, Respondents were asked questions regarding the frequency and quantity of consumption. These quantities were then standardized into cups by study staff, and fresh fruit and vegetable consumption was aggregated to develop variables for total fruit, total vegetable, and total fresh fruit and vegetable consumption of cups per day [42,51]. Additionally, outliers (values outside three standard deviations above the mean) were removed for the fresh fruit and vegetable consumption data.

Fresh fruit and vegetable purchasing also utilized the produce listed in the validated FRESH FFQ and was self-reported by program participants [42,51]. Participants reported the quantity via the number of items (i.e., one apple, two bananas, three serrano peppers, etc.) or pounds (i.e., five pounds of sweet potatoes) of fresh fruit and vegetables purchased and the frequency they purchased these items. This quantity was standardized to pounds by study staff using a protocol for each fruit and vegetable in the aforementioned list. These individual produce item quantities were aggregated and resulted in variables measuring total pounds of fresh fruit, total pounds of fresh vegetables, and total pounds of fresh fruit and vegetables. These values were then standardized to account for frequency and household size by developing total fresh fruit, total fresh vegetable, and total fresh fruit and vegetables purchased by the household in pounds per capita per week.

### 2.4. Covariates

Numerous demographic questions were included in the FRESH-Austin Baseline survey that were relevant for and used in the analysis. Race/ethnicity was self-reported by the participant in the survey. Urbanicity status for each respondent was determined by the zip code that they resided in and categorized based upon the Census definition and US Department of Defense definition of urban areas based on population density [53,54,55]. Zip codes with a population density of less than 1000 people per mile^2^ were categorized as rural, zip codes with a population density between 1000 and 3000 people per mile^2^ were categorized as peri-urban, and over 3000 people per mile^2^ were categorized as urban [54].

Income was self-reported and converted into a categorical variable, as under USD 25,000, between USD 25,000–44,999, USD 45,000–65,000, and over USD 65,000. Additionally, participation in a food assistance program (such as SNAP) was self-reported by the participant. Due to there being significant differences in the parent study in fresh fruit and vegetable consumption by recruitment arm, the recruitment arm was included as a covariate in the analyses [42].

### 2.5. Analysis Plan

#### Descriptive Statistics, Linear Regression, and Tests for Interactions

In this paper, we present findings from descriptive statistics, bivariate and multivariate linear regression models examining the association between fresh fruit and vegetable consumption and purchasing and geographic access to food, food insecurity status, and various demographic factors such as urbanicity, race/ethnicity, income, and utilization of food assistance. Additionally, in the adjusted model, the recruitment method is included as a control in the analysis due to significant differences in fresh fruit and vegetable consumption in the parent study. Furthermore, potential interactions will be assessed using Wald tests. These outliers were determined by if they were over three standard deviations above the mean. All statistical analyses were performed using Stata (version 16, College Station, TX, USA).

## 3. Results

### 3.1. Description of Sample

The final sample consisted of all participants that participated in the FRESH-Austin baseline survey that lived in Travis County and provided their address (N = 393). Descriptive data are presented in Table 1. Nearly 40% of participants reported being sometimes or often food insecure. The sample was predominantly Hispanic (54.10%) and resided in urban areas (55.38%), almost 23% of participants reported earning under USD 25,000 annually, and over 37% reported receiving food assistance in the last year. Over three-quarters of participants did not have a supermarket or large grocery store located within a 1500 m street network buffer of their home. On average, participants traveled over 5.25 miles to their reported utilized supermarket/large grocery store. Additionally, participants averaged consuming over 3.5 cups of fruits and vegetables per day and purchasing almost 8 pounds of fruits and vegetables per capita per week.

### 3.2. Fresh Fruit and Vegetable Consumption Findings

Bivariate and multivariate linear regression analyses were conducted in order to examine the association between geographic food access, food insecurity, and various covariates and fresh fruit and vegetable consumption, as presented in Table 2. In the unadjusted models, the presence of a supermarket within a 1500 m network buffer, race/ethnicity, and utilization of food assistance programs were significantly associated with fresh fruit and vegetable consumption. Specifically, individuals who had a supermarket within 1500 m of their home ate 0.44 cups more of fresh fruits and vegetables per day (*p* = 0.02) than those who did not have a supermarket located within 1500 m of their home. Additionally, participants who identified as Hispanic ate three-quarters of a cup more of fresh fruits and vegetables per day (*p* < 0.001) than non-Hispanic white participants. Lastly, individuals who used food assistance in the last year ate nearly half a cup (0.47) more of fresh fruits and vegetables per day (*p* = 0.01) than those who had not used food assistance in the last year.

However, after conducting Wald tests for interaction, there was a significant interaction between food insecurity status and race/ethnicity (*p* < 0.001) and fruit and vegetable consumption. Thus, this interaction needed to be accounted for in the adjusted model, which included the main exposures of interest of geographic access to food (presence of supermarket within 1500 m network buffer, and distance to utilized supermarket) and food insecurity status, as well as demographic factors of race/ethnicity, urbanicity, income, utilization of food assistance in last year, and recruitment arm of the study. In the adjusted findings, presence of a supermarket within 1500 m network buffer, and interaction terms for food insecurity and race/ethnicity were significantly associated with fresh fruit and vegetable consumption. Similar to the unadjusted findings, individuals who had a supermarket within 1500 m of their home ate 0.46 cups more of fruits and vegetables per day (*p* = 0.02) than those who did not have a supermarket located within 1500 m of their home. In the stratified food insecurity and race/ethnicity findings, food insecure non-Hispanic white participants ate less fruits and vegetables per day (β = −0.71, *p* = 0.04) than food secure non-Hispanic white participants. Additionally, food insecure Hispanic participants ate more fruits and vegetables per day (β = 0.60, *p* = 0.04) than food secure non-Hispanic white participants.

### 3.3. Fresh Fruit and Vegetable and Purchasing Findings

Similarly, bivariate and multivariate linear regression analyses were conducted in order to examine the association between geographic food access, food insecurity, and various covariates and fresh fruit and vegetable purchasing, as presented in Table 3. In the unadjusted models, distance to utilized supermarket was significantly associated with fresh fruit and vegetable purchasing. Specifically, individuals who traveled further to their self-reported utilized supermarket purchased less fruits and vegetables (β = −0.16, *p* = 0.02) than those who shopped close to home. Potential interactions were assessed using Wald tests; however, there were no significant interactions.

Similar to fresh fruit and vegetable consumption, the adjusted model included the main exposures of interest of geographic access to food (presence of supermarket within 1500 m network buffer, and distance to utilized supermarket) and food insecurity status, as well as demographic factors of race/ethnicity, urbanicity, income, utilization of food assistance in last year, and recruitment arm of the study. In the adjusted model, utilizing food assistance in the last year was associated with purchasing fewer fresh fruits and vegetables per capita per week (β = −1.83, *p* = 0.03) than those who were not on food assistance in the last year. No other factors were significantly associated.

## 4. Discussion

### 4.1. Summary of Findings

In summary, our study found that there were significant associations between fresh fruit and vegetable consumption and geographic access to food, food insecurity, and race/ethnicity, while fresh fruit and vegetable purchasing was associated with utilization of food assistance in the last year. Specifically, for fruit and vegetable consumption, there was a direct association between fruit and vegetable consumption and having a supermarket/large grocery store located within a 1500 m street network buffer of participants’ homes, compared to those that did not have a supermarket/large grocery store within 1500 m of their home. Of note, while not significant in the adjusted model, the indirect association between distance traveled to utilized supermarket/large grocery store and fruit and vegetable consumption was approaching significance, and future research should explore this relationship further. This association between the presence of a grocery store close to home and greater fruit and vegetable consumption has been found previously in other studies; however, this association with a racially/ethnically and socioeconomically diverse sample is a valued contribution to the literature [24,27].

Additionally, there was a significant interaction between food insecurity, race/ethnicity, and fresh fruit and vegetable consumption. Specifically, food insecure non-Hispanic white participants reported lower fresh fruit and vegetable consumption than food secure non-Hispanic white participants, and food insecure Hispanic participants having greater fresh fruit and vegetable consumption than food secure non-Hispanic white participants. This finding contributes to the mixed evidence found in the literature regarding the association between race/ethnicity and fruit and vegetable consumption. Some scholars have found that Hispanics living in the US have greater fruit and vegetable consumption than non-Hispanic whites in studies using nationally representative data and in contexts such as New York City [23,28,29]. However, this interaction between race/ethnicity, food insecurity status, and fruit and vegetable consumption validates the call by Houghtaling and colleagues (2022) and Singleton and collaborators (2020) for using an intersectional approach that accounts for various sociodemographic characteristics simultaneously when examining behaviors such as fruit and vegetable consumption and purchasing [8,10].

In terms of fresh fruit and vegetable purchasing behaviors, neither of the main independent variables of geographic access to food nor food insecurity status was associated with fresh fruit and vegetable purchasing behaviors in the adjusted model. However, in the adjusted model, utilization of food assistance in the last year was inversely associated with fresh fruit and vegetable purchasing. This could be due to the fact that there are often limited budgets for produce on food assistance plans such as Women, Infants and Children (WIC), or they could be receiving fresh produce through the charitable food system. However, more nuanced research is needed to explore those conjectures. Additionally, distance to utilized supermarket was inversely associated with fresh fruit and vegetable purchasing in the unadjusted model; however, this association was no longer significant in the adjusted model but was approaching significance. Therefore, this potential association between fresh fruit and vegetable purchasing behaviors and distance traveled to the utilized grocery store (rather than objectively measuring the distance to the closest grocery store) warrants future examination in additional settings and contexts. These findings are valuable contributions to the literature, given the limited research examining this topic.

### 4.2. Strengths and Limitations

#### 4.2.1. Strengths

There are numerous strengths to this study. One strength is that food insecurity status and geographic access to food are independent variables that are simultaneously examined in all analyses of this study. Additionally, this study adopted an intersectional approach for examining factors associated with fruit and vegetable consumption and purchasing, which as previously stated, has been highlighted by other scholars as needed in the literature. Finally, examining fruit and vegetable consumption and purchasing behaviors among this racially/ethnically diverse and lower-income sample provides tremendous insight into how these associations occur in populations of interest.

#### 4.2.2. Limitations

However, there are also limitations to this study. For instance, given the strategic and intentional sampling of racially/ethnically diverse, lower-income residents of Central Texas, this sample is not representative of the larger region or country. Additionally, these questions were only asked to the primary shopper of the household, therefore these behaviors may not be generalizable to other members of the household. Thus, there is limited generalizability. Additionally, we have employed a cross-sectional study design, therefore, the study lacks temporality and causality cannot be implied. Further, given this study relied on self-reported fresh fruit and vegetable consumption (although a valid instrument was used) and purchasing, there could be recall bias present systematically in the sample. However, despite these limitations, the relationships and associations examined in this study are a valuable and needed contribution to this literature base.

## 5. Conclusions

In conclusion, this study found that among a racially/ethnically and socioeconomically diverse sample, food insecurity status, geographic food access, and race/ethnicity were associated with fresh fruit and vegetable consumption, with a significant interaction between food insecurity status and race/ethnicity. Additionally, the main exposures of interest, food insecurity status and geographic food access, were not associated with fresh fruit and vegetable purchasing behaviors; however, participation in the food assistance program was inversely associated with fresh produce purchasing within this sample. These findings demonstrate the importance of taking an intersectional approach when examining fresh fruit and vegetable consumption and purchasing behaviors. Additionally, these findings suggest that communities experience food insecurity and limited healthy food access in different ways, and in some situations, are associated with healthy food consumption and purchasing behaviors. Thus, future research adopting an intersectionality-sensitive approach is needed to better understand how to best support under-served communities is needed.

## Figures and Tables

**Table 1:** Description of Sample.

N = 393	Variable	N	%	Mean	SD
**Presence of Supermarket within 1500 m Network Buffer**					
Supermarket Located within 1500 m		300	76.34		
No Supermarket within 1500 m		93	23.66		
**Distance to Utilized Supermarket (Continuous, in Miles)**					
Average Distance (in miles) to Self-Reported Utilized Supermarket				5.26	4.67
**Food Insecurity Status**					
Food Secure		236	60.20		
Sometimes/Often Food Insecure		156	39.80		
**Race/Ethnicity**					
Non-Hispanic White		126	32.31		
Hispanic		211	54.10		
Black/Other		53	13.59		
**Urbanicity**					
Urban		216	55.38		
Peri-Urban		92	23.59		
Rural		82	21.03		
**Income**					
Under USD 25,000		86	22.93		
USD 25,000–44,999		109	29.07		
USD 45,000–65,000		69	18.40		
USD 65,000+		111	29.60		
**Utilization of Food Assistance in Last Year**					
Has Received Food Assistance in Last Year		146	37.15		
Has Not Received Food Assistance in Last Year		247	62.85		
**Fresh Fruit and Vegetable Consumption (Cups/Day) and Purchasing (Pounds per Capita/Week)**					
Fresh Fruit and Vegetable Consumption (Cups/Day)				3.56	1.59
Fresh Fruit and Vegetable Purchasing (Pounds per Capita/Week)				7.99	6.20
**Recruitment Arm from Parent Study**					
Confirmed Users		123	31.30		
Geographically Exposed		185	47.07		
Comparison		85	21.63		

**Table 2:** Unadjusted and Adjusted Linear Regression Analytic Findings Exploring Associations with FV Consumption.

	Unadjusted			Adjusted		
	β	SE	*p*	β	SE	*p*
**Geographic Food Access Categorical (Referent = No Supermarket within 1500 m Network Buffer)**						
Presence of a Supermarket within 1500 m Network Buffer	**0.44**	**0.19**	**0.02**	**0.46**	**0.19**	**0.02**
**Distance to Utilized Supermarket (Continuous)**						
Distance to Utilized Supermarket	−0.01	0.02	0.42	−0.05	0.03	0.06
**Food Insecurity Status (Referent = Food Secure)**						
Sometimes/Often Food Insecure	0.14	0.16	0.40	-	-	-
**Race/Ethnicity (Referent = Non-Hispanic White)**						
Hispanic	**0.75**	**0.18**	**<0.001**	-	-	-
Black/Other	−0.23	0.25	0.35	-	-	-
**Urbanicity (Referent = Urban)**						
Peri-Urban	0.10	0.20	0.63	0.32	0.22	0.14
Rural	0.22	0.21	0.29	0.54	0.34	0.12
**Income (Referent = Under USD 25,000)**						
USD 25,000–45,000	0.12	0.23	0.60	0.16	0.23	0.47
USD 45,000–65,000	0.08	0.26	0.76	0.37	0.28	0.18
Over USD 65,000	−0.28	0.23	0.22	0.26	0.28	0.35
**Utilization of Food Assistance in Last Year (Referent = No)**						
Yes, used food assistance programs in last year	0.47	0.16	0.01	0.29	0.21	0.16
**Recruitment Arm of Study (Referent = Comparison)**						
Geographically Exposed	0.32	0.21	0.13	0.35	0.23	0.14
Confirmed Users	**0.56**	**0.22**	**0.01**	**0.65**	**0.25**	**0.01**
**Interaction between Food Insecurity Status and Race/Ethnicity (Referent = Food Secure and Non-Hispanic White)**						
Food Secure + Hispanic	-	-	-	0.34	0.25	0.18
Food Secure + Black/Other	-	-	-	−0.12	0.32	0.71
Food Insecure + Non-Hispanic White	-	-	-	**−0.71**	**0.35**	**0.04**
Food Insecure + Hispanic	-	-	-	**0.60**	**0.28**	**0.04**
Food Insecure + Black/Other	-	-	-	−0.72	0.42	0.09

**Table 3:** Unadjusted and Adjusted Linear Regression Analytic Findings Exploring Associations with FV purchasing (also adjusts for recruitment method).

	Unadjusted			Adjusted		
	β	SE	*p*	β	SE	*p*
**Geographic Food Access Categorical (Referent = No Supermarket within 1500 m Network Buffer)**						
Presence of a Supermarket within 1500 m Network Buffer	0.15	0.74	0.84	0.21	0.79	0.79
**Distance to Utilized Supermarket (Continuous)**						
Distance to Utilized Supermarket	**−0.16**	**0.07**	**0.02**	−0.16	0.12	0.18
**Food Insecurity Status (Referent = Food Secure)**						
Sometimes or Often Food Insecure	0.18	0.64	0.78	−0.09	0.78	0.91
**Race/Ethnicity (Referent = Non-Hispanic White)**						
Hispanic	0.74	0.70	0.29	0.93	0.86	0.28
Black/Other	0.69	1.02	0.50	1.38	1.12	0.22
**Urbanicity (Referent = Urban)**						
Peri-Urban	−0.09	0.77	0.91	0.19	0.92	0.83
Rural	−1.31	0.80	0.10	0.07	0.14	0.96
**Income (Referent = Under USD 25,000)**						
USD 25,000–45,000	0.28	0.91	0.76	−0.08	0.95	0.93
USD 45,000–65,000	−1.22	1.01	0.23	−1.65	1.14	0.14
Over USD 65,000	−1.37	0.90	0.13	−2.11	1.15	0.07
**Utilization of Food Assistance in Last Year (Referent = No)**						
Yes, used food assistance programs in last year	−0.51	0.65	0.43	**−1.83**	**0.85**	**0.03**
**Recruitment Arm of Study (Referent = Comparison)**						
Geographically Exposed	−0.45	0.81	0.58	0.41	0.97	0.67
Confirmed Users	0.58	0.88	0.51	1.00	1.03	0.33

## Data Availability

Data can be made available by request by contacting the authors.
